# Tris[2-eth­oxy-6-(methyl­imino­meth­yl)phenolato-κ^2^ *N*,*O* ^1^]cobalt(III) monohydrate

**DOI:** 10.1107/S1600536810040043

**Published:** 2010-10-13

**Authors:** Yin Dan Huang, Shu-Hua Zhang, Jiang Ke Qin, Fu Li Chen

**Affiliations:** a College of Chemistry and Bioengineering, Guilin University of Technology, Key Laboratory of Non-ferrous Metal Materials and Processing Technology, Ministry of Education, Guilin 541004, People’s Republic of China; b School of Chemistry and Chemical Engineering, Guangxi Normal University, Guilin 541004, People’s Republic of China

## Abstract

In the title compound, [Co(C_10_H_12_NO_2_)_3_]·H_2_O, the Co^III^ ion is coordinated by three O atoms and three N atoms from three bidentate 2-eth­oxy-6-(methyl­imino­meth­yl)phenolate ligands in a slightly distorted octa­hedral environment. The water mol­ecule connects two ligands by O—H⋯O hydrogen bonds. One terminal methyl group is disordered over two positions, with site-occupancy factors of 0.412 (15) and 0.588 (15).

## Related literature

For Co^III^ complexes, see: Park *et al.* (2008 ▶); Galezowski *et al.* (2008 ▶); Gupta *et al.* (2007 ▶). For Schiff-base compounds, see: Gupta & Sutar (2008 ▶); Sreenivasulu *et al.* (2005 ▶); Zhang & Feng (2010 ▶); Zhang *et al.* (2010 ▶).
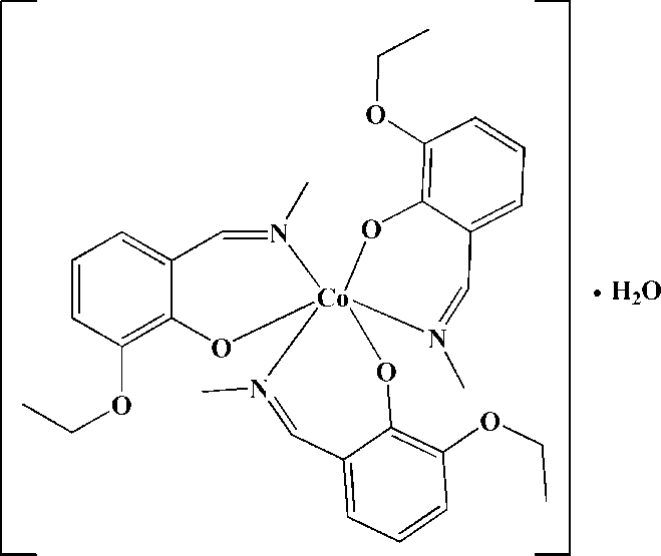


## Experimental

### 

#### Crystal data

[Co(C_10_H_12_NO_2_)_3_]·H_2_O
*M* *_r_* = 611.56
Monoclinic, 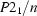

*a* = 14.022 (5) Å
*b* = 16.969 (6) Å
*c* = 14.233 (5) Å
β = 117.857 (4)°
*V* = 2994.1 (17) Å^3^
*Z* = 4
Mo *K*α radiation
μ = 0.62 mm^−1^
*T* = 296 K
0.42 × 0.20 × 0.09 mm

#### Data collection

Bruker SMART CCD area-detector diffractometer
Absorption correction: multi-scan (*SADABS*; Sheldrick, 1996 ▶) *T*_min_ = 0.860, *T*_max_ = 0.944
21931 measured reflections
5317 independent reflections
3929 reflections with *I* > 2σ(*I*)
*R*_int_ = 0.035

#### Refinement

*R*[*F* ^2^ > 2σ(*F* ^2^)] = 0.037
*wR*(*F* ^2^) = 0.100
*S* = 1.01
5317 reflections
379 parameters
12 restraints
H-atom parameters constrained
Δρ_max_ = 0.31 e Å^−3^
Δρ_min_ = −0.21 e Å^−3^

### 

Data collection: *SMART* (Bruker, 2004 ▶); cell refinement: *SAINT* (Bruker, 2004 ▶); data reduction: *SAINT*; program(s) used to solve structure: *SHELXS97* (Sheldrick, 2008 ▶); program(s) used to refine structure: *SHELXL97* (Sheldrick, 2008 ▶); molecular graphics: *SHELXTL* (Sheldrick, 2008 ▶); software used to prepare material for publication: *SHELXL97*.

## Supplementary Material

Crystal structure: contains datablocks global, I. DOI: 10.1107/S1600536810040043/bt5367sup1.cif

Structure factors: contains datablocks I. DOI: 10.1107/S1600536810040043/bt5367Isup2.hkl

Additional supplementary materials:  crystallographic information; 3D view; checkCIF report

## Figures and Tables

**Table 1:** Hydrogen-bond geometry (Å, °)

*D*—H⋯*A*	*D*—H	H⋯*A*	*D*⋯*A*	*D*—H⋯*A*
O1*W*—H1*WA*⋯O3	0.85	2.01	2.821 (3)	159
O1*W*—H1*WB*⋯O6	0.85	2.24	3.038 (3)	155

**Fig. 1.**
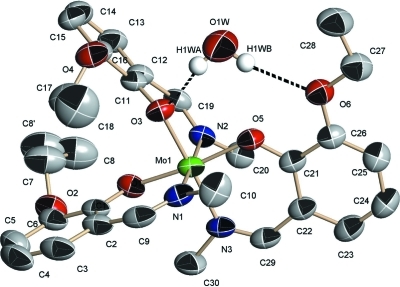
Molecular structure of the title compound with displacement ellipsoids drawn at the 50% probability level.

## supplementary crystallographic information

### Comment

Schiff base complexes have been studied for many years (Gupta & Sutar, 2008; Sreenivasulu *et al.*,2005; Zhang *et al.*, 2009; Zhang *et al.*, 2010) and have attracted   interest because of their anticancer, catalytic and fluorescent properties. Using 2-hydrogen-3-ethoxy-benzaldehyde, methylamine and Co(ClO_4_)_2_.6H_2_O, we have hydrothermally prepared the title compound, (I). As an example of Co^III^ compounds there is a series of Co^III^ complexes that were synthesized and characterized (Park *et al.* 2008; Galezowski *et al.* 2008; Gupta *et al.*, 2007).

In the molecular structure of (I), the Co^III^ ion is coordinated by three O atoms and three N atoms from three bidentate *L* ligand forming a slightly distorted octahedral geometry (Fig. 1). The Co–*X* (*X* = O, N) bond lengths lie in the range 1.878 (2)–1.954 (2) Å, and the angles subtended at the Co^III^ atom range from 85.61 (9) to 94.25 (10) °. The Co ion is in the 3+ oxidation state, as evidenced by bond valence summation calculations, charge balance considerations, and the presence of typical bond lengths for Co^III^ (Park, *et al.* 2008; Galezowski,*et al.* 2008; Gupta *et al.*, 2007). The water molecule connects two ligands by O–H···O hydrogen bonds (Table 1).

### Experimental

Complex I was prepared from a mixture of 2-hydrogen-3-ethoxy-benzaldehyde (0.166 g, 1 mmol), methylamine solution (0.5 ml), Co(ClO_4_)_2_.6H_2_O (0.360 g, 1 mmol), and methanol (8 ml) sealed in a 15 ml Teflon-lined stainless steel bomb, and kept at 120 °C for 72 h under autogenous pressure. After the reaction was slowly cooled to room temperature, red block crystals were produced (yield: 52%, based on 2-hydrogen-3-ethoxy-benzaldehyde). Anal. Calcd for C_30_H_38_CoN_3_O_7_ (%): C, 58.91; H, 6.26; N, 6.88. Found (%): C, 58.88; H, 6.31; N, 6.92.

### Refinement

H atoms were positioned geometrically and were treated as riding atoms, with C–H distances of 0.93–0.97 Å, O–H 0.85 Å with *U*_iso_(H) = 1.2 *U*_eq_(C_sp2_) and *U*_iso_(H) = 1.5*U*_eq_ (C_sp3_ or O). One terminal methyl group is disordered over two positions with site occupation factors of 0.412 (15) and 0.588 (15), respectively. The displacement parameters of the disordered atoms were restrained to an approximately isotropic behaviour.

### Crystal data

**Table:**

[Co(C_10_H_12_NO_2_)_3_]·H_2_O	*F*(000) = 1288
*M**_r_* = 611.56	*D*_x_ = 1.357 Mg m^−^^3^
Monoclinic, *P*2_1_/*n*	Mo *K*α radiation, λ = 0.71073 Å
Hall symbol: -P 2yn	Cell parameters from 7434 reflections
*a* = 14.022 (5) Å	θ = 2.8–28.4°
*b* = 16.969 (6) Å	µ = 0.62 mm^−^^1^
*c* = 14.233 (5) Å	*T* = 296 K
β = 117.857 (4)°	Block, red
*V* = 2994.1 (17) Å^3^	0.42 × 0.20 × 0.09 mm
*Z* = 4	

### Data collection

**Table:**

Bruker SMART CCD area-detector diffractometer	5317 independent reflections
Radiation source: fine-focus sealed tube	3929 reflections with *I* > 2σ(*I*)
graphite	*R*_int_ = 0.035
phi and ω scans	θ_max_ = 25.1°, θ_min_ = 2.8°
Absorption correction: multi-scan (*SADABS*; Sheldrick, 1996)	*h* = −16→16
*T*_min_ = 0.860, *T*_max_ = 0.944	*k* = −20→19
21931 measured reflections	*l* = −16→16

### Refinement

**Table:**

Refinement on *F*^2^	Primary atom site location: structure-invariant direct methods
Least-squares matrix: full	Secondary atom site location: difference Fourier map
*R*[*F*^2^ > 2σ(*F*^2^)] = 0.037	Hydrogen site location: inferred from neighbouring sites
*wR*(*F*^2^) = 0.100	H-atom parameters constrained
*S* = 1.01	*w* = 1/[σ^2^(*F*_o_^2^) + (0.0481*P*)^2^ + 0.9938*P*] where *P* = (*F*_o_^2^ + 2*F*_c_^2^)/3
5317 reflections	(Δ/σ)_max_ = 0.001
379 parameters	Δρ_max_ = 0.31 e Å^−^^3^
12 restraints	Δρ_min_ = −0.21 e Å^−^^3^

### Special details

**Table:**

Geometry. All e.s.d.'s (except the e.s.d. in the dihedral angle between two l.s. planes) are estimated using the full covariance matrix. The cell e.s.d.'s are taken into account individually in the estimation of e.s.d.'s in distances, angles and torsion angles; correlations between e.s.d.'s in cell parameters are only used when they are defined by crystal symmetry. An approximate (isotropic) treatment of cell e.s.d.'s is used for estimating e.s.d.'s involving l.s. planes.
Refinement. Refinement of *F*^2^ against ALL reflections. The weighted *R*-factor *wR* and goodness of fit *S* are based on *F*^2^, conventional *R*-factors *R* are based on *F*, with *F* set to zero for negative *F*^2^. The threshold expression of *F*^2^ > σ(*F*^2^) is used only for calculating *R*-factors(gt) *etc*. and is not relevant to the choice of reflections for refinement. *R*-factors based on *F*^2^ are statistically about twice as large as those based on *F*, and *R*- factors based on ALL data will be even larger.

### Fractional atomic coordinates and isotropic or equivalent isotropic displacement parameters (Å^2^)

**Table:**

	*x*	*y*	*z*	*U*_iso_*/*U*_eq_	Occ. (<1)
Co1	0.25696 (2)	0.943185 (18)	0.22915 (2)	0.04238 (12)	
C1	0.45768 (19)	0.96749 (15)	0.22521 (19)	0.0484 (6)	
C2	0.5048 (2)	0.89830 (16)	0.2845 (2)	0.0536 (7)	
C3	0.6109 (2)	0.87721 (19)	0.3075 (2)	0.0698 (9)	
H3	0.6412	0.8315	0.3461	0.084*	
C4	0.6695 (2)	0.9220 (2)	0.2748 (3)	0.0802 (10)	
H4	0.7388	0.9068	0.2896	0.096*	
C5	0.6255 (2)	0.9904 (2)	0.2193 (3)	0.0735 (9)	
H5	0.6666	1.0217	0.1983	0.088*	
C6	0.5225 (2)	1.01357 (16)	0.1942 (2)	0.0560 (7)	
C7	0.4056 (3)	1.0805 (2)	0.0383 (3)	0.0950 (12)	
H7A	0.3539	1.0397	0.0305	0.142*	
H7B	0.4399	1.0657	−0.0046	0.142*	
C9	0.4483 (2)	0.84859 (16)	0.3222 (2)	0.0583 (8)	
H9	0.4858	0.8048	0.3611	0.070*	
C10	0.3095 (3)	0.79479 (17)	0.3507 (3)	0.0754 (9)	
H10A	0.3647	0.7562	0.3871	0.113*	
H10B	0.2486	0.7701	0.2931	0.113*	
H10C	0.2874	0.8174	0.3994	0.113*	
C11	0.19289 (18)	0.90745 (15)	0.01213 (19)	0.0451 (6)	
C12	0.14933 (18)	0.98317 (15)	−0.02230 (19)	0.0469 (6)	
C13	0.1192 (2)	1.00754 (18)	−0.1260 (2)	0.0583 (7)	
H13	0.0903	1.0576	−0.1475	0.070*	
C14	0.1313 (2)	0.9596 (2)	−0.1968 (2)	0.0673 (8)	
H14	0.1114	0.9768	−0.2655	0.081*	
C15	0.1737 (2)	0.88476 (19)	−0.1645 (2)	0.0624 (8)	
H15	0.1816	0.8515	−0.2124	0.075*	
C16	0.2044 (2)	0.85881 (16)	−0.0627 (2)	0.0541 (7)	
C17	0.3562 (3)	0.7792 (2)	0.0374 (3)	0.0882 (11)	
H17A	0.3757	0.8204	0.0905	0.132*	
H17B	0.3972	0.7870	−0.0010	0.132*	
C18	0.3805 (4)	0.7025 (3)	0.0884 (4)	0.1320 (17)	
H18A	0.3397	0.6952	0.1264	0.198*	
H18B	0.4561	0.6993	0.1372	0.198*	
H18C	0.3617	0.6621	0.0355	0.198*	
C19	0.12739 (19)	1.03513 (14)	0.0451 (2)	0.0484 (6)	
H19	0.0843	1.0787	0.0125	0.058*	
C20	0.1228 (2)	1.08673 (15)	0.1956 (2)	0.0552 (7)	
H20A	0.1819	1.1201	0.2407	0.083*	
H20B	0.0950	1.0604	0.2374	0.083*	
H20C	0.0669	1.1181	0.1420	0.083*	
C21	0.1177 (2)	0.90195 (14)	0.32031 (19)	0.0456 (6)	
C22	0.1746 (2)	0.95016 (15)	0.4104 (2)	0.0514 (7)	
C23	0.1439 (3)	0.95312 (17)	0.4916 (2)	0.0644 (8)	
H23	0.1814	0.9858	0.5500	0.077*	
C24	0.0613 (3)	0.90943 (19)	0.4861 (2)	0.0690 (8)	
H24	0.0411	0.9128	0.5396	0.083*	
C25	0.0061 (2)	0.85931 (16)	0.4001 (2)	0.0616 (7)	
H25	−0.0501	0.8286	0.3971	0.074*	
C26	0.0336 (2)	0.85452 (15)	0.3196 (2)	0.0504 (6)	
C27	−0.0963 (3)	0.75265 (17)	0.2315 (2)	0.0699 (8)	
H27A	−0.1573	0.7824	0.2271	0.105*	
H27B	−0.0671	0.7213	0.2960	0.105*	
C28	−0.1311 (3)	0.7007 (2)	0.1376 (3)	0.0891 (11)	
H28A	−0.1621	0.7320	0.0741	0.134*	
H28B	−0.1838	0.6639	0.1360	0.134*	
H28C	−0.0699	0.6724	0.1418	0.134*	
C29	0.2648 (2)	0.99735 (16)	0.4233 (2)	0.0556 (7)	
H29	0.2972	1.0275	0.4849	0.067*	
C30	0.3961 (2)	1.05901 (18)	0.3891 (3)	0.0714 (9)	
H30A	0.4084	1.0867	0.4526	0.107*	
H30B	0.3782	1.0961	0.3323	0.107*	
H30C	0.4602	1.0307	0.4012	0.107*	
N1	0.35163 (18)	0.85684 (12)	0.30894 (16)	0.0514 (5)	
N2	0.16059 (15)	1.02795 (11)	0.14483 (16)	0.0435 (5)	
N3	0.30595 (16)	1.00281 (12)	0.35992 (17)	0.0505 (5)	
O1	0.36036 (12)	0.99193 (9)	0.19967 (13)	0.0493 (4)	
O2	0.48376 (15)	1.08425 (12)	0.14429 (17)	0.0684 (6)	
O3	0.21754 (13)	0.87875 (9)	0.10683 (12)	0.0486 (4)	
O4	0.24247 (16)	0.78279 (11)	−0.03486 (15)	0.0663 (5)	
O5	0.13858 (13)	0.89719 (9)	0.23996 (13)	0.0475 (4)	
O6	−0.01521 (15)	0.80540 (10)	0.23346 (14)	0.0584 (5)	
O1W	0.0519 (2)	0.76823 (16)	0.0638 (2)	0.1173 (10)	
H1WA	0.1112	0.7907	0.0777	0.176*	
H1WB	0.0468	0.7664	0.1214	0.176*	
C8	0.3302 (13)	1.1506 (9)	0.0252 (13)	0.110 (3)	0.412 (15)
H8A	0.2703	1.1327	0.0347	0.165*	0.412 (15)
H8B	0.3041	1.1727	−0.0446	0.165*	0.412 (15)
H8C	0.3693	1.1901	0.0775	0.165*	0.412 (15)
C8'	0.3642 (9)	1.1540 (6)	−0.0134 (9)	0.110 (3)	0.588 (15)
H8'A	0.4168	1.1790	−0.0287	0.165*	0.588 (15)
H8'B	0.3487	1.1874	0.0323	0.165*	0.588 (15)
H8'C	0.2993	1.1451	−0.0784	0.165*	0.588 (15)

### Atomic displacement parameters (Å^2^)

**Table:**

	*U*^11^	*U*^22^	*U*^33^	*U*^12^	*U*^13^	*U*^23^
Co1	0.04589 (19)	0.03229 (19)	0.03721 (19)	0.00247 (14)	0.00959 (14)	−0.00092 (14)
C1	0.0427 (14)	0.0450 (15)	0.0415 (14)	0.0012 (11)	0.0063 (11)	−0.0116 (11)
C2	0.0462 (14)	0.0468 (16)	0.0471 (15)	0.0083 (12)	0.0045 (12)	−0.0074 (13)
C3	0.0526 (17)	0.0628 (19)	0.065 (2)	0.0147 (15)	0.0036 (15)	−0.0126 (16)
C4	0.0423 (16)	0.089 (3)	0.088 (2)	0.0107 (17)	0.0131 (17)	−0.022 (2)
C5	0.0472 (16)	0.082 (2)	0.082 (2)	−0.0067 (16)	0.0221 (15)	−0.0183 (19)
C6	0.0468 (15)	0.0523 (17)	0.0553 (17)	−0.0053 (12)	0.0123 (13)	−0.0132 (13)
C7	0.091 (3)	0.080 (2)	0.079 (3)	−0.006 (2)	0.010 (2)	0.005 (2)
C9	0.0626 (18)	0.0465 (16)	0.0420 (15)	0.0183 (14)	0.0046 (13)	−0.0014 (12)
C10	0.101 (2)	0.0520 (18)	0.077 (2)	0.0271 (17)	0.0448 (19)	0.0278 (16)
C11	0.0425 (13)	0.0451 (14)	0.0374 (14)	−0.0065 (11)	0.0101 (11)	−0.0028 (12)
C12	0.0384 (12)	0.0487 (15)	0.0412 (14)	−0.0022 (11)	0.0082 (11)	0.0031 (12)
C13	0.0508 (15)	0.0639 (18)	0.0447 (16)	−0.0012 (13)	0.0095 (13)	0.0111 (14)
C14	0.0594 (17)	0.089 (2)	0.0431 (16)	−0.0056 (16)	0.0151 (14)	0.0104 (16)
C15	0.0616 (17)	0.078 (2)	0.0435 (16)	−0.0102 (15)	0.0213 (14)	−0.0110 (15)
C16	0.0540 (15)	0.0520 (17)	0.0472 (16)	−0.0055 (12)	0.0161 (13)	−0.0054 (13)
C17	0.097 (3)	0.070 (2)	0.082 (2)	0.0242 (19)	0.029 (2)	−0.0117 (19)
C18	0.146 (4)	0.102 (3)	0.136 (4)	0.039 (3)	0.055 (3)	0.041 (3)
C19	0.0393 (13)	0.0409 (14)	0.0512 (17)	0.0029 (10)	0.0097 (12)	0.0071 (12)
C20	0.0560 (15)	0.0399 (14)	0.0639 (18)	0.0072 (12)	0.0232 (14)	−0.0006 (13)
C21	0.0536 (14)	0.0365 (14)	0.0390 (14)	0.0087 (11)	0.0151 (12)	0.0041 (11)
C22	0.0596 (16)	0.0447 (15)	0.0424 (15)	0.0068 (12)	0.0177 (13)	−0.0027 (12)
C23	0.080 (2)	0.0571 (18)	0.0532 (18)	0.0083 (15)	0.0281 (16)	−0.0088 (14)
C24	0.090 (2)	0.067 (2)	0.0576 (19)	0.0080 (18)	0.0416 (17)	−0.0022 (16)
C25	0.0729 (19)	0.0503 (17)	0.0662 (19)	0.0057 (14)	0.0364 (16)	0.0074 (15)
C26	0.0601 (16)	0.0382 (14)	0.0457 (15)	0.0055 (12)	0.0186 (13)	0.0061 (12)
C27	0.0747 (19)	0.0583 (19)	0.070 (2)	−0.0154 (15)	0.0278 (16)	0.0028 (16)
C28	0.095 (3)	0.076 (2)	0.083 (2)	−0.0341 (19)	0.031 (2)	−0.0142 (19)
C29	0.0616 (17)	0.0471 (16)	0.0433 (16)	0.0008 (13)	0.0122 (14)	−0.0111 (12)
C30	0.0659 (18)	0.069 (2)	0.067 (2)	−0.0190 (15)	0.0213 (15)	−0.0273 (16)
N1	0.0646 (14)	0.0390 (12)	0.0384 (12)	0.0099 (10)	0.0137 (10)	0.0026 (9)
N2	0.0417 (11)	0.0325 (11)	0.0474 (13)	−0.0011 (8)	0.0132 (10)	0.0001 (9)
N3	0.0500 (12)	0.0426 (12)	0.0448 (12)	−0.0006 (10)	0.0102 (10)	−0.0085 (10)
O1	0.0423 (9)	0.0417 (10)	0.0525 (10)	0.0045 (7)	0.0126 (8)	0.0039 (8)
O2	0.0624 (12)	0.0585 (12)	0.0731 (14)	−0.0073 (10)	0.0224 (11)	−0.0016 (11)
O3	0.0635 (11)	0.0344 (9)	0.0394 (10)	0.0011 (8)	0.0170 (8)	−0.0007 (7)
O4	0.0811 (14)	0.0505 (12)	0.0580 (12)	−0.0003 (10)	0.0247 (11)	−0.0132 (9)
O5	0.0579 (10)	0.0390 (10)	0.0387 (9)	−0.0051 (8)	0.0167 (8)	−0.0030 (8)
O6	0.0678 (12)	0.0476 (11)	0.0572 (11)	−0.0114 (9)	0.0270 (10)	−0.0023 (9)
O1W	0.137 (2)	0.126 (2)	0.1031 (19)	−0.0782 (19)	0.0675 (18)	−0.0525 (17)
C8	0.108 (5)	0.083 (3)	0.097 (5)	0.009 (4)	0.013 (3)	0.016 (4)
C8'	0.108 (5)	0.083 (3)	0.097 (5)	0.009 (4)	0.013 (3)	0.016 (4)

### Geometric parameters (Å, °)

**Table:**

Co1—O1	1.8792 (18)	C17—H17B	0.9700
Co1—O5	1.9044 (18)	C18—H18A	0.9600
Co1—O3	1.9061 (17)	C18—H18B	0.9600
Co1—N3	1.940 (2)	C18—H18C	0.9600
Co1—N1	1.947 (2)	C19—N2	1.276 (3)
Co1—N2	1.954 (2)	C19—H19	0.9300
C1—O1	1.305 (3)	C20—N2	1.468 (3)
C1—C2	1.416 (4)	C20—H20A	0.9600
C1—C6	1.416 (4)	C20—H20B	0.9600
C2—C3	1.411 (4)	C20—H20C	0.9600
C2—C9	1.423 (4)	C21—O5	1.309 (3)
C3—C4	1.352 (5)	C21—C22	1.412 (3)
C3—H3	0.9300	C21—C26	1.424 (4)
C4—C5	1.376 (5)	C22—C23	1.411 (4)
C4—H4	0.9300	C22—C29	1.434 (4)
C5—C6	1.374 (4)	C23—C24	1.347 (4)
C5—H5	0.9300	C23—H23	0.9300
C6—O2	1.370 (3)	C24—C25	1.391 (4)
C7—O2	1.391 (4)	C24—H24	0.9300
C7—C8	1.546 (16)	C25—C26	1.371 (4)
C7—C8'	1.427 (10)	C25—H25	0.9300
C7—H7A	0.9700	C26—O6	1.372 (3)
C7—H7B	0.9700	C27—O6	1.437 (3)
C9—N1	1.286 (3)	C27—C28	1.481 (4)
C9—H9	0.9300	C27—H27A	0.9700
C10—N1	1.463 (4)	C27—H27B	0.9700
C10—H10A	0.9600	C28—H28A	0.9600
C10—H10B	0.9600	C28—H28B	0.9600
C10—H10C	0.9600	C28—H28C	0.9600
C11—O3	1.318 (3)	C29—N3	1.281 (3)
C11—C12	1.409 (3)	C29—H29	0.9300
C11—C16	1.415 (4)	C30—N3	1.480 (3)
C12—C13	1.395 (4)	C30—H30A	0.9600
C12—C19	1.438 (4)	C30—H30B	0.9600
C13—C14	1.365 (4)	C30—H30C	0.9600
C13—H13	0.9300	O1W—H1WA	0.8507
C14—C15	1.386 (4)	O1W—H1WB	0.8550
C14—H14	0.9300	C8—H8A	0.9600
C15—C16	1.377 (4)	C8—H8B	0.9600
C15—H15	0.9300	C8—H8C	0.9600
C16—O4	1.381 (3)	C8'—H8'A	0.9600
C17—O4	1.439 (4)	C8'—H8'B	0.9600
C17—C18	1.452 (5)	C8'—H8'C	0.9600
C17—H17A	0.9700		
			
O1—Co1—O5	172.32 (7)	H18A—C18—H18C	109.5
O1—Co1—O3	88.46 (8)	H18B—C18—H18C	109.5
O5—Co1—O3	86.96 (7)	N2—C19—C12	127.1 (2)
O1—Co1—N3	91.17 (9)	N2—C19—H19	116.5
O5—Co1—N3	93.87 (9)	C12—C19—H19	116.5
O3—Co1—N3	175.49 (8)	N2—C20—H20A	109.5
O1—Co1—N1	94.28 (9)	N2—C20—H20B	109.5
O5—Co1—N1	91.54 (9)	H20A—C20—H20B	109.5
O3—Co1—N1	85.92 (8)	N2—C20—H20C	109.5
N3—Co1—N1	89.63 (9)	H20A—C20—H20C	109.5
O1—Co1—N2	85.59 (8)	H20B—C20—H20C	109.5
O5—Co1—N2	88.43 (8)	O5—C21—C22	124.5 (2)
O3—Co1—N2	92.24 (8)	O5—C21—C26	118.8 (2)
N3—Co1—N2	92.21 (9)	C22—C21—C26	116.7 (3)
N1—Co1—N2	178.15 (9)	C23—C22—C21	120.3 (3)
O1—C1—C2	124.5 (3)	C23—C22—C29	117.6 (3)
O1—C1—C6	118.5 (2)	C21—C22—C29	122.1 (3)
C2—C1—C6	117.0 (2)	C24—C23—C22	121.1 (3)
C3—C2—C1	119.8 (3)	C24—C23—H23	119.5
C3—C2—C9	118.3 (3)	C22—C23—H23	119.5
C1—C2—C9	121.9 (2)	C23—C24—C25	119.8 (3)
C4—C3—C2	121.4 (3)	C23—C24—H24	120.1
C4—C3—H3	119.3	C25—C24—H24	120.1
C2—C3—H3	119.3	C26—C25—C24	121.0 (3)
C3—C4—C5	119.4 (3)	C26—C25—H25	119.5
C3—C4—H4	120.3	C24—C25—H25	119.5
C5—C4—H4	120.3	C25—C26—O6	124.4 (3)
C6—C5—C4	121.6 (3)	C25—C26—C21	121.0 (3)
C6—C5—H5	119.2	O6—C26—C21	114.6 (2)
C4—C5—H5	119.2	O6—C27—C28	108.2 (3)
O2—C6—C5	119.9 (3)	O6—C27—H27A	110.1
O2—C6—C1	119.2 (2)	C28—C27—H27A	110.1
C5—C6—C1	120.7 (3)	O6—C27—H27B	110.1
O2—C7—C8	104.0 (6)	C28—C27—H27B	110.1
O2—C7—C8'	116.3 (5)	H27A—C27—H27B	108.4
O2—C7—H7A	108.9	C27—C28—H28A	109.5
C8—C7—H7A	95.8	C27—C28—H28B	109.5
C8'—C7—H7A	117.5	H28A—C28—H28B	109.5
O2—C7—H7B	108.9	C27—C28—H28C	109.5
C8—C7—H7B	129.5	H28A—C28—H28C	109.5
C8'—C7—H7B	96.0	H28B—C28—H28C	109.5
H7A—C7—H7B	107.9	N3—C29—C22	127.6 (2)
N1—C9—C2	128.0 (2)	N3—C29—H29	116.2
N1—C9—H9	116.0	C22—C29—H29	116.2
C2—C9—H9	116.0	N3—C30—H30A	109.5
N1—C10—H10A	109.5	N3—C30—H30B	109.5
N1—C10—H10B	109.5	H30A—C30—H30B	109.5
H10A—C10—H10B	109.5	N3—C30—H30C	109.5
N1—C10—H10C	109.5	H30A—C30—H30C	109.5
H10A—C10—H10C	109.5	H30B—C30—H30C	109.5
H10B—C10—H10C	109.5	C9—N1—C10	117.4 (2)
O3—C11—C12	123.8 (2)	C9—N1—Co1	123.5 (2)
O3—C11—C16	119.1 (2)	C10—N1—Co1	118.94 (19)
C12—C11—C16	117.0 (2)	C19—N2—C20	117.0 (2)
C13—C12—C11	120.4 (3)	C19—N2—Co1	122.78 (18)
C13—C12—C19	118.1 (2)	C20—N2—Co1	120.17 (17)
C11—C12—C19	121.4 (2)	C29—N3—C30	117.1 (2)
C14—C13—C12	121.6 (3)	C29—N3—Co1	124.30 (18)
C14—C13—H13	119.2	C30—N3—Co1	118.6 (2)
C12—C13—H13	119.2	C1—O1—Co1	127.84 (17)
C13—C14—C15	118.9 (3)	C6—O2—C7	116.3 (2)
C13—C14—H14	120.6	C11—O3—Co1	123.20 (15)
C15—C14—H14	120.6	C16—O4—C17	113.3 (2)
C16—C15—C14	121.1 (3)	C21—O5—Co1	127.20 (15)
C16—C15—H15	119.4	C26—O6—C27	117.1 (2)
C14—C15—H15	119.4	H1WA—O1W—H1WB	107.7
C15—C16—O4	119.3 (3)	C7—C8—H8A	109.5
C15—C16—C11	121.0 (3)	C7—C8—H8B	109.5
O4—C16—C11	119.6 (2)	H8A—C8—H8B	109.5
O4—C17—C18	108.6 (3)	C7—C8—H8C	109.5
O4—C17—H17A	110.0	H8A—C8—H8C	109.5
C18—C17—H17A	110.0	H8B—C8—H8C	109.5
O4—C17—H17B	110.0	C7—C8'—H8'A	109.5
C18—C17—H17B	110.0	C7—C8'—H8'B	109.5
H17A—C17—H17B	108.4	H8'A—C8'—H8'B	109.5
C17—C18—H18A	109.5	C7—C8'—H8'C	109.5
C17—C18—H18B	109.5	H8'A—C8'—H8'C	109.5
H18A—C18—H18B	109.5	H8'B—C8'—H8'C	109.5
C17—C18—H18C	109.5		

### Hydrogen-bond geometry (Å, °)

**Table:**

*D*—H···*A*	*D*—H	H···*A*	*D*···*A*	*D*—H···*A*
O1W—H1WA···O3	0.85	2.01	2.821 (3)	159
O1W—H1WB···O6	0.85	2.24	3.038 (3)	155
